# Effects of folic acid on oxidative damage of kidney in lead-exposed rats

**DOI:** 10.3389/fnut.2022.1035162

**Published:** 2022-11-15

**Authors:** Ning Li, Liuding Wen, Zengli Yu, Tiange Li, Tianlin Wang, Mingwu Qiao, Lianjun Song, Xianqing Huang

**Affiliations:** ^1^Henan Engineering Technology Research Center of Food Processing and Circulation Safety Control, College of Food Science and Technology, Henan Agricultural University, Zhengzhou, China; ^2^College of Public Health, Zhengzhou University, Zhengzhou, China

**Keywords:** folic acid, lead, kidney, oxidative stress, endoplasmic reticulum stress

## Abstract

**Introduction:**

Lead (Pb) has many applications in daily life, but in recent years, various problems caused by lead exposure have aroused people’s concern. Folic acid is widely found in fruits and has received more attention for its antioxidant function. However, the role of folic acid in lead-induced kidney injury in rats is unclear. This study was designed to investigate the effects of folic acid on oxidative stress and endoplasmic reticulum stress in the kidney of rats caused by lead exposure.

**Methods:**

Forty specific pathogen-free male *Rattus norvegicus* rats were randomly divided into control, lead, intervention, and folic acid groups. The levels of SOD, GSH-Px, GSH, and MDA were measured by biochemical kits. The protein levels of Nrf2, HO-1, CHOP, and GRP78 were measured by immunofluorescence.

**Results:**

This study showed that lead exposure increased the blood levels of lead in mice. However, the intervention of folic acid decreased the levels of lead, but the difference was not statistically significant. Lead exposure causes oxidative stress by decreasing kidney SOD, GSH-Px, and GSH levels and increasing MDA levels. However, folic acid alleviated the oxidative damage caused by lead exposure by increasing the levels of GSH-Px and GSH and decreasing the levels of MDA. Immunofluorescence results showed that folic acid intervention downregulated the upregulation of kidney Nrf2, HO-1, GRP78, and CHOP expression caused by lead exposure.

**Discussion:**

Overall, folic acid alleviates kidney oxidative stress induced by lead exposure by regulating Nrf2 and HO-1, while regulating CHOP and GRP78 to mitigate apoptosis caused by excessive endoplasmic reticulum stress.

## Graphical Abstract

It illustrates the mechanism of action of folic acid in alleviating oxidative stress and endoplasmic reticulum stress in the kidney caused by lead exposure. 
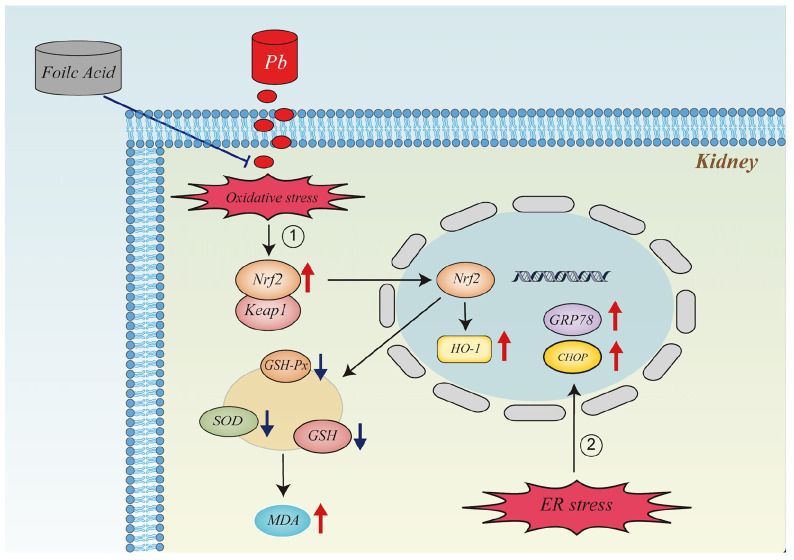

## Introduction

Lead (Pb) is widely distributed in nature and used in human life, but it is also a highly toxic pollutant (1). Although people are aware of its hazards and have tried to control the exposure levels of lead in the environment. Nevertheless, lead is non-biodegradable and accumulates in the body, which causes lead poisoning to remain one of the world’s most common health problems (2–4). Lead can cause various physiological, biochemical and behavioral disorders in the central and peripheral nervous system, hematopoietic system, cardiovascular system, kidney, liver and reproductive system, and destroy the integrity of the biological mechanism of oxidative stress (1, 5). As one of the main excretion pathways of lead in organisms, the kidney is the target organ of lead cytotoxicity.

Oxidative stress is a key factor in the mechanism of lead actors (6, 7). Chronic lead exposure generates excess reactive oxygen species (ROS) and different free radicals. These free radicals promote the high production of reactive oxygen species and then the production of malondialdehyde (MDA) (8). At the same time, the body’s antioxidant systems are damaged, such as superoxide dismutase (SOD), catalase (CAT), and glutathione (GSH) redox systems (8). The nuclear factor erythroid 2-related factor 2 (Nrf2)/heme oxygenase 1 (HO-1) signaling pathway is thought to be a major defense mechanism for oxidative stress-induced renal cytotoxicity (9, 10). Nrf2 is an oxidative stress-related transcription factor that can exert antioxidant effects by entering the nucleus to activate HO-1 (11, 12).

The endoplasmic reticulum (ER) is one of the important subcellular organelles in cells. Its function mainly enables the protein to be correctly folded, assembled, and transported after being synthesized, modified, and processed (13). Several factors can lead to dysregulation of the ER microenvironment, such as hypoxia, nutrient imbalance, reactive oxygen species, and low PH. All these factors can lead to protein misfolding and accumulation of unfolded proteins, thus causing ER stress (13). When cells are subjected to ER stress, a protective response is initiated accordingly. The unfolded protein response (UPR) is a stress-protective response. It can reduce and alleviate the burden and damage of the ER to some extent, restore the proteostasis of ER and re-establish ER homeostasis (14–16). However, if the UPR is insufficient to restore and maintain ER homeostasis when subjected to more intense and prolonged stress, it can lead to apoptosis (17). C/EBP-homologous protein (CHOP) and glucose-regulated protein 78 (GPR78) are key markers of ER stress, and their upregulation indicates that cells suffer from different levels of ER stress (18). It has been shown that lung cancer cell death is caused by ER stress, which may be due to ER stress-mediated apoptosis (19). In addition, it has also been found that lead-induced nephrotoxicity is mainly due to apoptosis, which is closely associated with ER stress (20).

Oxidative stress and ER stress are interactive (21). When cells undergo oxidative stress, the redox homeostasis of the ER is disrupted, which disturbs the function of the ER and finally leads to ER stress. However, ER stress also generates a large number of reactive oxygen species, further aggravating oxidative stress. The downstream signaling pathways of ER stress and oxidative stress overlap. ER stress can activate the Nrf2 signaling pathway through protein kinase RNA-like ER kinase (PERK) (22, 23). It has been shown that curcumin can effectively upregulate the level of reactive oxygen species in lung cancer cells, which in turn activates ER stress, leading to apoptosis and cell scorching (19).

Folic acid is a water-soluble B-type vitamin, which plays a key biological role in many physiological processes, especially in single carbon transfer reactions, nucleic acid synthesis, and methionine regeneration (24, 25). There is evidence that folic acid regulates lipid metabolism and oxidative stress, scavenges ROS, inhibits the activity of ROS-producing enzymes, and restores the activity of antioxidant enzymes. It is an effective free radical scavenger (26). In addition, by its antioxidant action, folic acid can inhibit the activation of the nuclear factor kappa-light-chain-enhancer of activated B cells (NF-κB) (27).

Currently, there is less report on the repairing effect of folic acid on oxidative damage of rat kidneys caused by lead exposure. Therefore, we designed a lead-exposed rat model to study the effects of folic acid on the antioxidant indexes and the expression of Nrf2, HO-1, GRP78, and CHOP in the kidneys of lead-exposed rats and to explore the repair effect of folic acid on lead-induced oxidative damage in the kidneys of rats.

## Materials and methods

### Animals and treatments

Two months old SPF grade SD male rats were acquired from Henan Laboratory Animal Center (SYXK 2018-0005). The rats were housed in a clean and sterile environment with a temperature of 18–22°C, 12/12 light/dark, and humidity of 50–60%. After a week of adaptive feeding, start the experiment (Irradiation experimental pellet feed provided by the Experimental Animal Center of Henan Province).

The doses of lead and folic acid used in this study were the same as those previously reported (28, 29). Forty rats were randomly divided into four groups: Control group (*n* = 10), free drinking water, and 1 mL deionized water was intragastrical administered. Lead group (*n* = 10), free drink 0.2% lead acetate solution and 1 mL deionized water were intragastrical administered. Intervention group (*n* = 10), free drink 0.2% lead acetate solution, and 1 mL folic acid (Sigma-Aldrich) suspension was intragastrical administered (prepared according to the dose of 0.4 mg/kg BW folic acid). Folic acid group (*n* = 10), free drinking water and 1 mL folic acid suspension were intragastrical administered (prepared according to the dose of 0.4 mg/kg BW folic acid).

Two weeks later, the rats were anesthetized with 10% chloral hydrate. After fixation, kidney tissues were dissected, and the indexes were determined. The Scientific Ethics Committee approved the experiment, and the experimental process was carried out according to the operating rules of animal experiments. The scientific Ethics Committee approved the experimental animal protocol of Henan Agricultural University (Zhengzhou, China) (Ethical protocol code: 4101055342743).

### Determination of blood lead content

The determination of lead concentration in blood was consistent with previous studies (30). Briefly, 100 μl of blood sample was mixed with 3,900 μl of ultrapure nitric acid and centrifuged for 10 min, and the supernatant was analyzed for lead concentration. The blood lead content was determined by Z-5000 graphite furnace atomic absorption spectrometer (Hitachi, Ltd., Japan). Parameters: wavelength 283.3 nm, passband 0.5 nm, lamp current 9 mA, 95°C 15 s, 105°C 15 s, 800°C 15 s, 2500°C 3 s.

### Determination of kidney oxidative stress indicators

The measurement of oxidative stress indicators was consistent with previous studies (31). In brief, 10% tissue homogenate was prepared according to rat tissue weight (g): 0.86% physiological saline volume (mL) = 1:9. The prepared 10% tissue homogenate was centrifuged at 4,000 rpm for 10 min at 4°C and the centrifuged tissue homogenate was discarded. According to the experiment, an appropriate amount of the supernatant is diluted with physiological saline to a suitable concentration for measurement. The activity of SOD, GSH-Px, and the content of GSH and MDA in the kidney were determined by the kit produced by Nanjing Jiancheng Institute of Bioengineering.

### Immunofluorescence

The measurement of immunofluorescence was consistent with previous studies (32). In brief, the kidney tissue fixed with 4% paraformaldehyde solution was embedded in the slice and then dewaxed to water. The sections were microwaved in citrate buffer (PH = 6) for 10 min, and 3% H_2_O_2_ blocked the endogenous peroxidase at room temperature for 20 min. 3% of goat serum was added dropwise to block endogenous biotin at room temperature for 20 min and then incubated with primary antibody at 4°C overnight. The primary antibodies were rabbit polyclonal antibody-Nrf2 (1:200; Proteintech, China), rabbit polyclonal antibody-HO-1 (1:100; Proteintech, China), rabbit polyclonal antibody-CHOP (1:200; Proteintech, China), rabbit polyclonal antibody-GRP78 (1:200; Proteintech, China). The second antibody was added and incubated at 37°C for 2 h. DAPI stained the nucleus for 1 min and sealed the tablet with anti-fluorescence quenching. The images were collected under the fluorescence microscope. Before each step, soak in PBS solution for 5 min and clean three times.

### Statistical analysis

All experimental data were expressed as mean ± SD, and One-way ANOVA analyzed data by Duncan’s test for multiple comparisons using SPSS 23.0 software (IBM Corporation, Armonk, NY, United States). *P* < 0.05 was considered a significant difference.

## Results

### Effect of folic acid on serum lead content of lead-exposed rats

**FIGURE 1:**
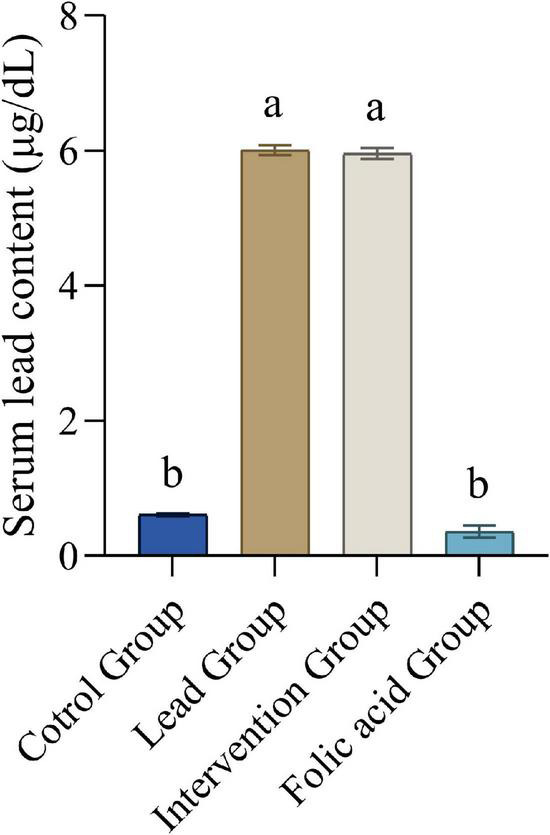
Effect of folic acid on serum lead content of lead-exposed rats (*n* = 10). Data were presented as mean ± SD. Different superscript letters indicate differences (*P* < 0.05).

Figure 1 shows the blood lead content of each group of rats. It was evident that compared with the control group, the blood lead content in the lead group and the intervention group was significantly increased (*P* < 0.05), and the blood lead content in the folic acid group was decreased, but the difference was not statistically significant (*P* > 0.05). Compared with the lead group, the blood lead content of the intervention group decreased, but the difference was not statistically significant (*P* > 0.05), and the blood lead content of the folic acid group was significantly decreased (*P* < 0.05).

### Effects of folic acid on superoxide dismutase, GSH-Px activity and glutathione, malondialdehyde content in kidneys of lead-exposed rats

**FIGURE 2:**
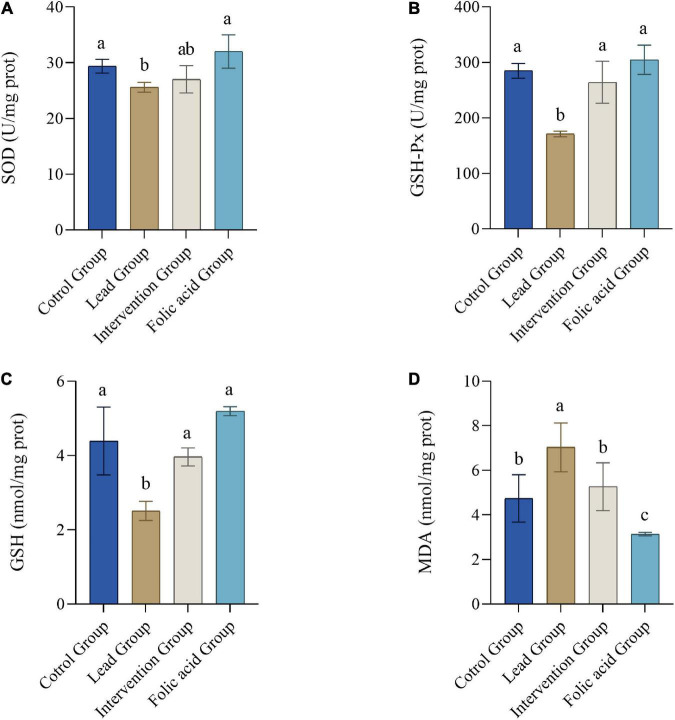
Effects of folic acid on superoxide dismutase (SOD) **(A)**, GSH-Px **(B)** activities and glutathione (GSH) **(C)**, malondialdehyde (MDA), **(D)** contents in kidney of lead-exposed rats (*n* = 10). Data were presented as mean ± SD. Different superscript letters indicate differences (*P* < 0.05).

Figure 2 shows the changes of SOD, GSH-Px activity and GSH, MDA contents in kidney tissues of rats in each group. In the lead group, SOD, GSH-Px activity and GSH content in the kidney decreased significantly compared with the control, but MDA content increased significantly (*P* < 0.05). However, compared to the lead group, the intervention group elevated the activity of GSH-Px and GSH levels and decreased the level of MDA, with statistically significant differences (*P* < 0.05). Although the activity of SOD was also elevated, there was no statistical difference (*P* > 0.05). Compared with the control group, the folic acid group increased the activity of SOD and GSH-Px and the level of GSH, but the difference was not statistically significant (*P* > 0.05). At the same time, the levels of MDA were reduced, and the difference was statistically significant (*P* < 0.05).

### Effect of folic acid on the expression of nuclear factor erythroid 2-related factor 2 protein in the kidney of lead exposed rats

**FIGURE 3:**
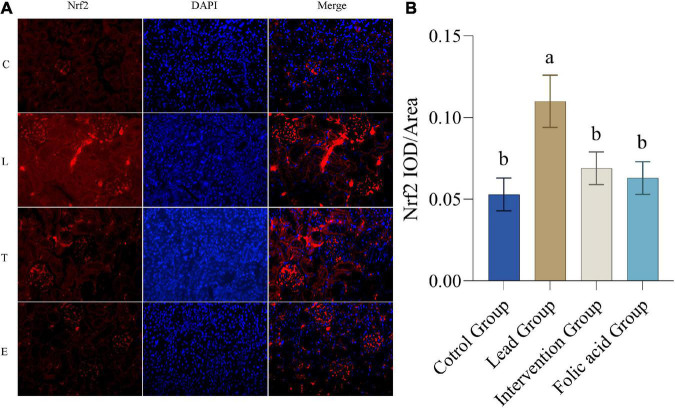
Effect of folic acid on the expression of nuclear factor erythroid 2-related factor 2 (Nrf2) protein in the kidney of lead exposed rats. **(A)** Immunofluorescence results of Nrf2 protein expression (200×). **(B)** The expression level of Nrf2 protein is represented by the average integrated optical density value (*n* = 6). C: control group; L: lead-exposed group; T: intervention group; E: folic acid group. Data were presented as mean ± SD. Different superscript letters indicate differences (*P* < 0.05).

Expression of Nrf2 protein in the kidney was detected by immunofluorescence. The results of immunofluorescence are shown in Figure 3A. As can be seen by Figure 3B, the expression of NRF2 was up-regulated in the lead group compared with the control group, and the difference was statistically significant (*P* < 0.05). The expression of NRF2 was down-regulated in the intervention group compared with the lead group, and the difference was statistically significant (*P* < 0.05).

### Effect of folic acid on the expression of heme oxygenase 1 protein in the kidney of lead exposed rats

**FIGURE 4:**
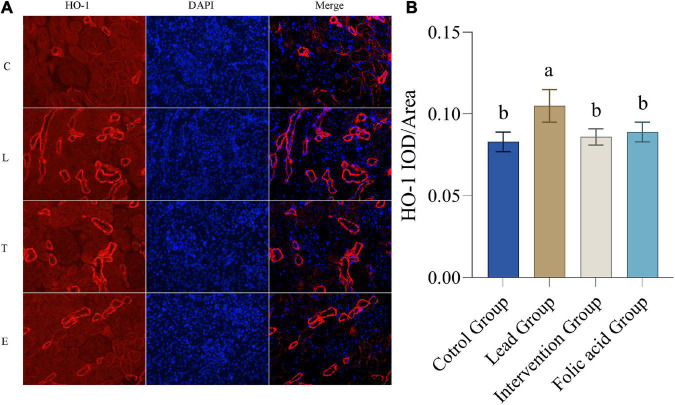
Effect of folic acid on the expression of heme oxygenase 1 (HO-1) protein in the kidney of lead exposed rats. **(A)** Immunofluorescence results of HO-1 protein expression (200×). **(B)** The expression level of HO-1 protein is represented by the average integrated optical density value (*n* = 6). C: control group; L: lead-exposed group; T: intervention group; E: folic acid group. Data were presented as mean ± SD. Different superscript letters indicate differences (*P* < 0.05).

The expression of HO-1 protein in the kidney was detected by immunofluorescence. The results of immunofluorescence are shown in Figure 4A. As can be seen by Figure 4B, the expression of HO-1 was up-regulated in the lead group compared with the control group, and the difference was statistically significant (*P* < 0.05). The expression of HO-1 was down-regulated in the intervention group compared with the lead group and returned to the level of the control group, and the difference was statistically significant (*P* < 0.05).

### Effect of folic acid on the expression of C/EBP-homologous protein in the kidney of lead exposed rats

**FIGURE 5:**
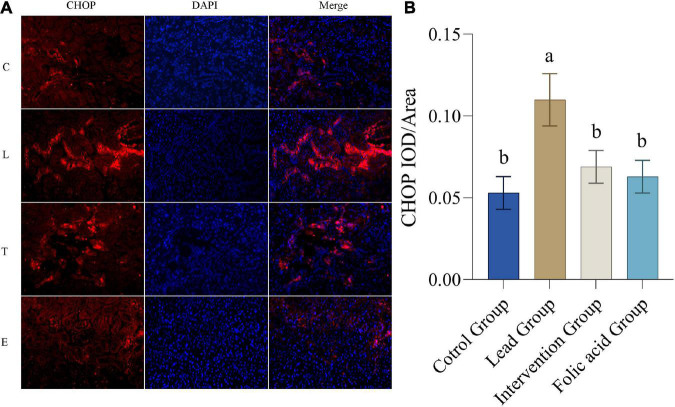
Effect of folic acid on the expression of C/EBP-homologous protein (CHOP) protein in the kidney of lead exposed rats. **(A)** Immunofluorescence results of CHOP protein expression (200×). **(B)** The expression level of CHOP protein is represented by the average integrated optical density value (*n* = 6). C: control group; L: lead-exposed group; T: intervention group; E: folic acid group. Data were presented as mean ± SD. Different superscript letters indicate differences (*P* < 0.05).

The expression of CHOP protein in the kidney was detected by immunofluorescence. The results of immunofluorescence are shown in Figure 5A. As shown in Figure 5B, the expression of CHOP was upregulated in the lead group compared with the control group, and the difference was statistically significant (*P* < 0.05). Compared with the lead group, the expression of CHOP in the intervention group was down-regulated and returned to the level of the control group, and the difference was statistically significant (*P* < 0.05).

### Effect of folic acid on the expression of glucose-regulated protein 78 in the kidney of lead exposed rats

**FIGURE 6:**
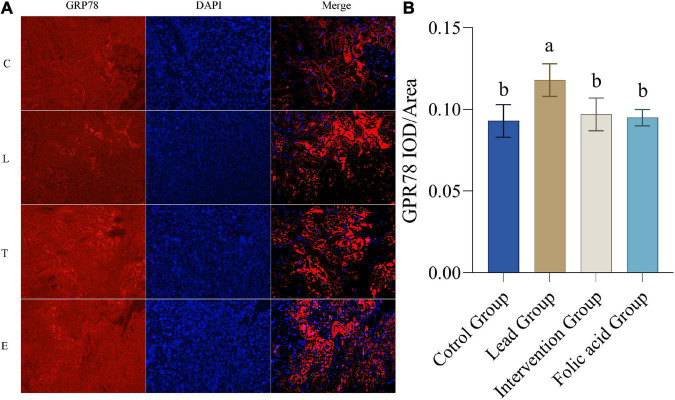
Effect of folic acid on the expression of glucose-regulated protein 78 (GRP78) protein in the kidney of lead exposed rats. **(A)** Immunofluorescence results of GRP78 protein expression (200×). **(B)** The expression level of GRP78 protein is represented by the average integrated optical density value (*n* = 6). C: control group; L: lead-exposed group; T: intervention group; E: folic acid group. Data were presented as mean ± SD. Different superscript letters indicate differences (*P* < 0.05).

The expression of GRP78 protein in the kidney was detected by immunofluorescence. The results of immunofluorescence are shown in Figure 6A. As shown in Figure 6B, the expression of GRP78 was upregulated in the lead group compared to the control group, and the difference was statistically significant (*P* < 0.05). Compared with the lead group, the expression of CHOP in the intervention group was down-regulated and returned to the level of the control group, and the difference was statistically significant (*P* < 0.05).

## Discussion

Lead enters the blood and quickly accumulates in soft tissues such as the brain, liver, and kidneys and is most common in the kidneys (7, 33). Lead-induced toxicity is mainly manifested in the production of reactive oxygen species, inhibition of SOD, GSH-Px, and other antioxidant enzymes, disruption of the oxidation/antioxidant balance *in vivo*, ER stress, and mitochondrial damage (3, 34). Folic acid, as a vitamin, can participate in DNA synthesis as a methyl donor and plays a key role in preventing single- and double-stranded DNA breaks. In recent years, folic acid has been confirmed to enhance neuroplasticity and antioxidant function (35). Some studies have shown that folic acid can alleviate lipid disorders in rats fed with a high-fat diet, enhance the ability of antioxidant defense and enhance the activity of antioxidant enzymes (36). Therefore, this study hypothesized that folic acid treatment could alleviate the oxidative stress caused by lead exposure.

In this study, the extent of lead exposure was determined by measuring lead concentrations in the blood of mice. The antioxidant capacity of folic acid was verified by measuring SOD, GSH-Px, GSH, and MDA in the kidney tissues of mice. Finally, the effect of folic acid on the expression of antioxidant-related proteins Nrf2, HO-1, and ER stress-related proteins CHOP and GRP78 in mouse kidneys was examined by immunofluorescence.

Previous studies have shown that lead treatment increases lead levels in the blood of mice (30). By measuring lead concentrations in the blood of mice, this study found a 10-fold increase in blood lead levels in mice in the lead-exposed group compared to the control group, which is consistent with previous findings. Through further experiments, it was found that the lead levels in the blood of mice in the intervention group were reduced, but the difference was not statistically significant, this may be because lead has a very stable structure and is not easily degraded (37). There was no difference between the blood levels of lead in the mice treated with folic acid alone and the control group, demonstrating that folic acid treatment alone does not increase the levels of lead in the blood of mice.

The kidney is the body’s main organ for accumulation and excretion and is also a target organ for lead exposure (38). Many studies have shown that lead exposure causes cell oxidative stress (39–41). When cells are subjected to oxidative stress, large amounts of ROS and lipid peroxides are produced, and the antioxidant system is damaged. It has been reported that hydrogen peroxide-induced oxidative stress in cells causes an increase in the levels of ROS and MDA and a decrease in SOD and GSH (42). Vitamin C in combination with vitamin E has been reported to alleviate oxidative stress caused by lead exposure by increasing the levels of SOD, CAT, and GSH-Px (43, 44). The present study found that lead exposure significantly increased MDA levels and significantly decreased SOD, GSH, and GSH-Px simultaneously. Therefore, this suggests that lead exposure induced oxidative stress in the kidney. The folic acid intervention of significantly increased the levels of GSH and GSH-Px and significantly decreased the levels of MDA. The levels of SOD also increased, but there was no statistical difference; this may be because folic acid regulates the antioxidant system by upregulating GSH and GSH-Px, causing a decrease in MDA levels. However, SOD may not be involved in this process.

It is well known that Nrf2 is a key factor in the endogenous antioxidant system and plays an important role in cell antioxidants and against exogenous damage (39). After being transferred to the nucleus, Nrf2 binds to antioxidant response elements (ARE), regulates the expression of HO-1, SOD, and other enzymes, and enhances the antioxidant defense ability (45). HO-1 is one of the important antioxidant enzymes regulated by Nrf2. It is an important part of the antioxidant system in the body and plays a vital role in the oxidative stress damage of cells (46). There is evidence that the Nrf2/HO-1 signaling pathway protects nerves in a rat model of Parkinson’s disease induced by 1-methyl-4-phenyl-1,2,3,6-tetrahydropyridine (MPTP) and also improves lipopolysaccharide Induced acute lung injury (47). In recent years, the role of Nrf2/HO-1 in kidney oxidative stress injury has received increasing attention. Studies have shown that the formation of kidney stones can be suppressed through the Nrf2/HO-1 signaling pathway (48). In this study, the expression of Nrf2 was highest in the lead exposure group, approximately one-fold compared with the control group. The expression of HO-1 was also the highest in the lead exposure group. This indicated that the Nrf2/HO-1 signaling pathway was activated under the lead exposure condition. After the folic acid intervention, the expression of Nrf2 and HO-1 decreased, indicating that folic acid can repair the oxidative damage caused by lead exposure to the kidneys. However, the mechanism of folic acid in alleviating oxidative damage caused by lead exposure is still unclear, and further research is needed.

As previously reported, apoptosis is involved in lead-induced nephrotoxicity, which is closely related to ER stress (2). Furthermore, it is noteworthy that ER stress may damage cells by activating apoptosis, which may result from excessive ER stress (49). Related studies have shown that ER stress is involved in the development of renal fibrosis and contributes to the development of chronic kidney disease by promoting apoptosis in renal tubular cells (20). GRP78 and CHOP are considered to be markers of ER stress (50). Excessive ER stress causes apoptosis by activating CHOP. ROS-mediated ER stress has been reported to be attenuated by inhibition of CHOP protein (51). In addition, it was also illustrated that LBP could protect HaCat cells from PM2.5-induced apoptosis and toxicity by decreasing the expression of CHOP and GRP78 (52). The results of the present study showed that the expression of GRP78 and CHOP proteins was significantly increased in the lead-exposed group compared to the control group, indicating that lead induces ER stress, which is consistent with previous studies. Meanwhile, the present study showed that the expression of GRP78 and CHOP decreased after folic acid intervention compared with the lead group, suggesting that folic acid could mitigate the damage caused by lead exposure by inhibiting ER stress and could be restored to normal levels.

## Conclusion

In the present study, folic acid reduced the blood lead levels in lead-exposed mice, but there was no statistical difference. Also, folic acid alleviated oxidative stress caused by lead exposure by increasing kidney GSH and GSH-Px levels, thereby reducing MDA production. Lead exposure upregulated kidney Nrf2, HO-1, CHOP, and GRP78 expression. However, folic acid intervention reversed this regulation. The results of this study suggest that folic acid alleviates oxidative stress and ER stress caused by lead exposure in the kidney by regulating the expression of Nrf2, HO-1, CHOP, and GRP78.

## Data availability statement

The raw data supporting the conclusions of this article will be made available by the authors, without undue reservation.

## Ethics statement

The animal study was reviewed and approved by the Scientific Ethics Committee of Henan Agricultural University (Zhengzhou, China).

## Author contributions

NL designed the whole experiment. NL and LW conducted the experiments and wrote the manuscript. TL and TW analyzed the data. ZY, XH, LS, and MQ provided the technical support and assistance. LW revised the manuscript. All authors read and approved the manuscript.

## Abbreviations

Pb: lead
ROS: reactive oxygen species
GSH: glutathione
MDA: malondialdehyde
Nrf2: nuclear factor erythroid 2-related factor 2
HO-1: heme oxygenase 1
ARE: anti-oxidative response element
SOD: superoxide dismutase
ER: endoplasmic reticulum
UPR: unfolded protein response
GRP78: glucose-regulated protein 78
PERK: protein kinase RNA-like ER kinase
CHOP: C/EBP-homologous protein.
